# Primary osteosarcoma of the breast after complete resection of a metaplastic ossification: a case report

**DOI:** 10.1186/s13256-016-1008-2

**Published:** 2016-08-17

**Authors:** Evariste Gafumbegete, Uta Fahl, Regina Weinhardt, Michael Respondek, Alaa Eldin Elsharkawy

**Affiliations:** 1 Department of Pathology, Ludmillenstift hospital, Meppen, Germany; 2 Department of gynecology and obstetrics, Ludmillenstift hospital, Meppen, Germany; 3 Clinic of Emsland-gynecology, Lingen, Germany; 4 Clinic of pathology, Vechta, Germany; 5 Neurosurgery Teaching Program, Faculty of Medicine, University of Traditional Medicine, Yerevan, Armenia

**Keywords:** Primary osteosarcoma, Metaplastic ossification, Ductal hyperplasia

## Abstract

**Background:**

Primary osteosarcoma of the breast is an extremely rare lesion. The pathogenesis of primary osteosarcomas is controversial.

**Case presentation:**

We present the case of a 63-year-old white German woman who presented with a mass in her right breast after routine screening. The core needle biopsy showed ductal hyperplasia with metaplastic ossification of the breast tissue. Complete excision of the lesion with standard safety margins was performed. The final diagnosis was metaplastic ossification. Three years later, our patient presented again with a painless lump in her right breast about 15 × 8 × 7 cm, extending to the lower part of axilla with skin ulceration. Pathologic diagnosis was osteosarcoma. Positron emission tomography and computed tomography and staging showed no other lesions. Modified radical mastectomy and axillary lymph node dissection was performed, no lymph node metastases were found.

**Conclusions:**

Our case highlights the possibility that primary osteosarcoma of the breast may develop after complete resection with the classical safety margin for metaplastic ossification. Long-term follow-up after resection of this benign breast lesion is required.

## Background

Breast sarcomas are very uncommon and make up less than 1 % of all primary breast malignancies. Primary osteosarcoma accounts for about 12.5 % of all breast sarcomas.

The histogenesis of primary osteosarcoma of the breast is unknown, prognosis and optimal treatment remain uncertain because of the rarity of this tumor. Published data consist almost exclusively of single case reports, with one major series [1]. More data and information regarding this rare tumor are highly needed. We present a rare case of primary osteosarcoma that developed 3 years after complete resection with the safety margin of metaplastic ossification.

## Case presentation

A 63-year-old white German woman presented with a lump in her right breast following routine screening for breast cancer.

**Fig. 1:**
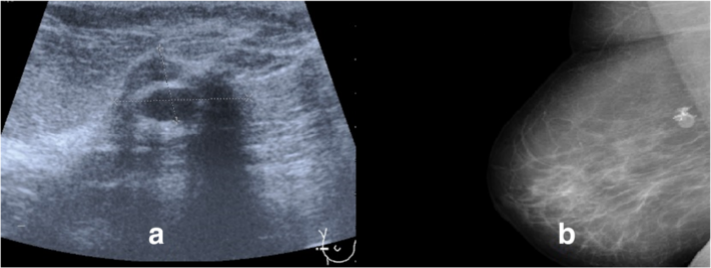
**a** Sonographic picture of the breast showing an irregular lesion, 11.1 × 20.1 mm. **b** Mammography showed a calcified prepectoral fine delimited mass

Screening mammography demonstrated a very dense calcified 35-mm lobular mass in her medial right breast. An ultrasound scan showed an irregular lesion 11.1 × 20.1 mm (Fig. 1).

A clinical examination of her breast revealed a golf-ball-sized mass located at the 6–9-o’clock position in her right breast, arterial hypertension, otherwise no other symptoms or neurological deficits were present.

**Fig. 2:**
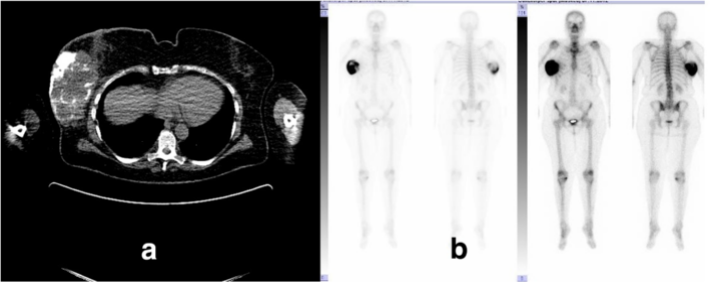
**a** Computed tomography scan of the chest demonstrates a calcifying tumor mass of the right breast. No other tumor in chest or elsewhere was seen. **b** Scintigraphy shows a huge tumor in the right breast with calcification areas. No others tumors at the bone site were seen

**Fig. 3:**
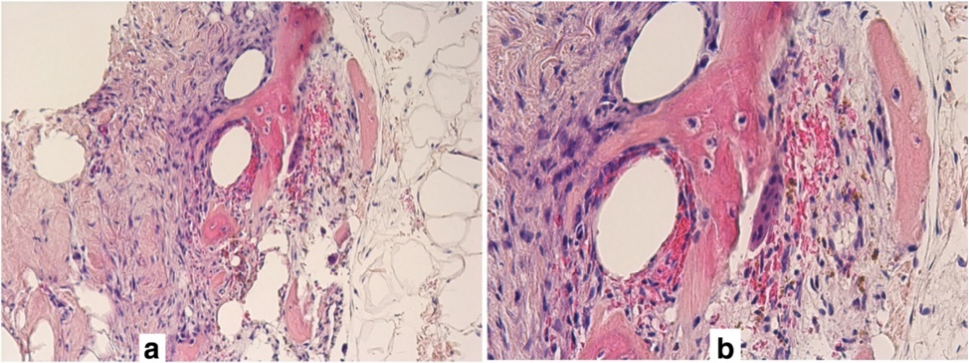
**a** in the middle of the picture newly formed bone trabeculae with delimited fat tissue on the right and fibrous tissue on the left. Core biopsy, Hematoxylin and eosin, 60×. **b** Magnification of Fig. 3a showing benign bone tissue with an example of a trabecula in the middle surrounded by osteoclasts to the right and osteoblasts to the left. In between osteocytes, no evidence of malignancy. Hematoxylin and eosin,100×

Intervention: in July 2009, a core needle biopsy of the lesion was performed, histologically showing ductal hyperplasia with metaplastic ossification of breast tissue. No malignant tissue was detected. The specimens were examined by two different pathologists of two different institutes. As differential diagnosis, a metaplastic carcinoma was considered. Complete excision was recommended by the pathologist. In October 2009, complete excision of the lesion with standard safety margins was performed. The diagnosis was metaplastic ossification. Again Figs. 2 and 3, the specimen was examined by two different pathologists. No postoperative therapy was recommended. Follow-up examinations at intervals of 3–6 months were recommended; after 2 years of uneventful follow-up further examinations were refused by our patient.

**Fig. 4:**
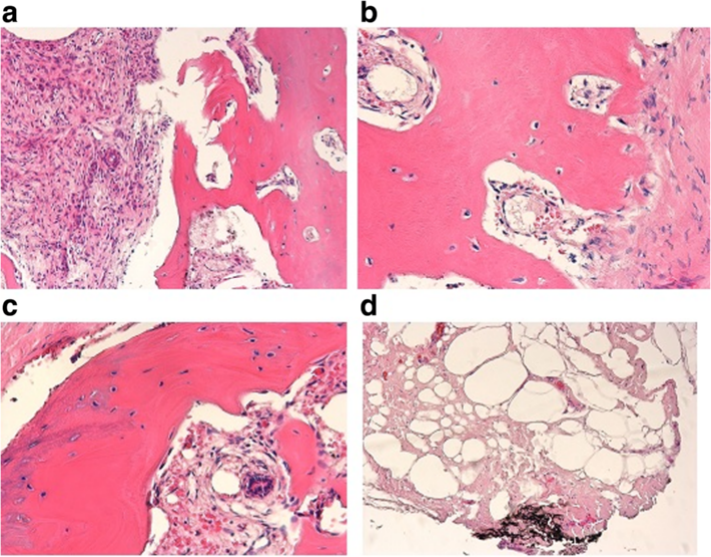
**a-d** Excision specimen, histological pictures. **a** Benign lesion after the core biopsy has been gained. The resorptive connective tissue to the left is seen. Hematoxylin and eosin, 40×. **b** and **c**: like (**a**). Hematoxylin and eosin, 60×. **d** Tumor-free resection margin. The ink is seen at the bottom. Hematoxylin and eosin, 10×

One year later our patient presented again with a lump in her right breast about 150 × 80 × 70 mm, extending to the lower part of the axilla with skin ulceration. Positron emission tomography and computed tomography (PET-CT) and staging showed no further metastases (Fig. 4).

**Fig. 5:**
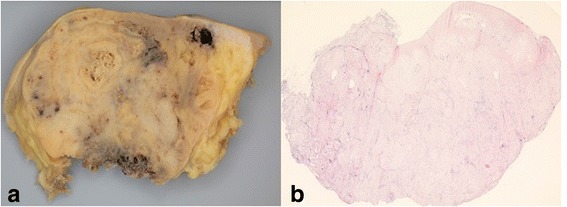
**a** Macroscopy of an osteosarcoma of the breast. Yellowish destructive aggressive growth of the tumor ulcerating the skin. Alveolar bone formation seen at the bottom, in the middle and at the upper right of the picture. **b** Histology osteosarcoma of the breast in whole scale image. Hematoxylin and eosin, 1×

**Fig. 6:**
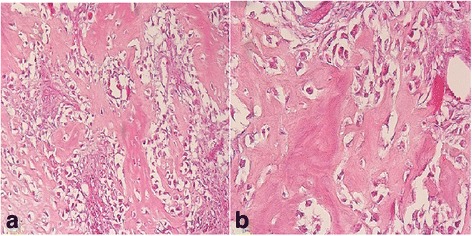
**a** Aggressive tumor cells destroying the connective tissue between the trabeculae of the bone tumor. Hematoxylin and eosin, 60×. **b** Magnification of tumor cells and trabeculae of the bone tissue as demonstrated in Fig. 6a Hematoxylin and eosin, 100×

Pathological examination: the tumor specimen was fixed in 4 % phosphate-buffered formalin and routinely paraffin-embedded. Sections 4 μm thick were stained with hematoxylin and eosin as well as Elastica van Gieson.

Moreover they were immunostained with the avidin biotin-peroxidase complex method using the following antibodies: anti-high molecular epithelial marker (cytokeratins: ck 5/6 and ck pan: AE 1/3), anti-EMA (cytomed, 1:100).

Immunohistochemically, the tumor did not express any epithelial tissue markers at all. Anti-EMA reaction showed no gland development in the tumor mass.

The case with all specimens went again to Canada for a second opinion (Figs 5 and 6). The first diagnosis had been confirmed. No further pathological examination was planned.

Surgery and postoperative follow-up: in November 2012, modified radical mastectomy and axillary lymph node dissection were performed, no lymph node metastases were found. Postoperative chemotherapy followed. PET-CT and staging showed no evidence of metastatic or recurrent disease. One year after surgery our patient died of complications due to lung metastases and pleural effusion.

## Discussion

Primary osteosarcomas of the breast are extremely rare lesions with few published data. Histogenesis, prognosis and optimal treatment of primary osteosarcoma remain uncertain [1], and is controversial; an origin from normal breast tissue de novo or as osseous metaplasia in a pre-existing benign or malignant neoplasm of the breast or as non-phylloides sarcoma has been suggested [2, 3].

Primary osteosarcoma should be diagnosed only after metaplastic mammary carcinoma is ruled out. However, metaplastic carcinoma shows an epithelial differentiation. Therefore, it is essential to examine a major number of tumor tissue blocks, also by means of immunohistochemistry [1]. In our case, the tumor showed neoplastic bone and cartilage formation with no evidence of epithelial differentiation. The pathological examination was performed both in Germany and in Canada.

The primary osteosarcoma presented here developed in breast tissue following surgical removal of the metaplastic ossification with safety margins, which microscopically were found to be tumor-free. Based on the existing data, we may presume that the primary osteosarcoma described here arose from normal breast tissue, but the presence of the previously completely excised metaplastic ossification makes it difficult to prove this assumption. Our case report is in agreement with previously published data [4–7].

Our case highlights the importance of close follow-ups in cases of benign breast lesions such as metaplastic ossifications. Primary osteosarcoma should be included routinely in the differential diagnosis of breast tumors. Initially, our patient had clinically been diagnosed as having breast carcinoma. In almost every case in the literature, the diagnosis of primary osteocarcoma was established histologically [8].

Primary osteosarcoma of the breast occurs in a wide range of age, from 12 to 89 years, predominantly in middle-aged and older women [1, 2]. The tumors vary in size up to maximum diameters of 30 cm and may be smooth or lobulated, solid or cystic, freely mobile or fixed and occasionally ulcerated. No specific clinical features reliably distinguish it from other malignant breast tumors [1].

Breast osteosarcomas are highly malignant, most patients dying within 2 years. It spreads mainly by the hematogenous route and axillary nodes are rarely involved. Tumor response to adjuvant chemotherapy is unclear. Surgery remains the most favored therapy, some authors recommend simple mastectomy as the treatment of choice, as wide local excision may be complicated by early local recurrence [9]. However, others recommend a wide surgical excision of the tumor without axillary node dissection [1]. Adjuvant radiotherapy is recommended as an addition to the treatment, although no standard of care for these lesions has been made [10].

## Conclusions

It is important to be aware of this unusual occurrence and to differentiate it from metaplastic carcinoma, as well as from malignant phylloides tumor, as it has a different behavior and prognosis.

## Abbreviations

PET-CT, positron emission tomography and computed tomography
